# Cheap Eating

**Published:** 1858-02

**Authors:** 


### CHEAP EATING.

THREE and a half pounds of corn (Indian) meal, a handful of salt, one teaspoonful, or not (we would prefer the *not*), of carbonate of soda; mix well, and pour over it a sufficient amount of *boiling* water to soften the whole, then pour on a quart of cold water, sprinkle over it three-quarters of a pound of dry flour, and stir it well. Divide into five puddings; put each into a floured cloth, tie tight, put into boiling water, and boil three hours; eat it hot, or cold, or fried. It is said that this will give a family of twelve persons two hearty meals, at a cost of twenty-five cents. Eat it with syrup.

The *gambrel*, or "vein piece" of a "beef," is the lower part of the muscle of the hind leg; it has no bone in it, and sells for eight cents a pound, when a steak or roasting-piece costs eighteen cents a pound. Three pounds of this, boiled most thoroughly, will make soup enough for a family of eight persons, for two dinners; and if the meat itself is set away cold, it will make a pleasant relish for two or three minor meals besides. There is no waste in it, and all for twenty-five cents.

---

NEVER talk with a pin in your mouth. Never walk through a garden, field, or wood, chewing a splinter, or straw, or blade of grass. Never go to church with dilapidated soles, they are very conspicuous to those who walk behind.

